# Reviewing the essential roles of remote phenotyping, GWAS and explainable AI in practical marker-assisted selection for drought-tolerant winter wheat breeding

**DOI:** 10.3389/fpls.2024.1319938

**Published:** 2024-04-18

**Authors:** Ignacio Chang-Brahim, Lukas J. Koppensteiner, Lorenzo Beltrame, Gernot Bodner, Anna Saranti, Jules Salzinger, Phillipp Fanta-Jende, Christoph Sulzbachner, Felix Bruckmüller, Friederike Trognitz, Mina Samad-Zamini, Elisabeth Zechner, Andreas Holzinger, Eva M. Molin

**Affiliations:** ^1^ Unit Bioresources, Center for Health & Bioresources, AIT Austrian Institute of Technology, Tulln, Austria; ^2^ Saatzucht Edelhof GmbH, Zwettl, Austria; ^3^ Unit Assistive and Autonomous Systems, Center for Vision, Automation & Control, AIT Austrian Institute of Technology, Vienna, Austria; ^4^ Department of Crop Sciences, Institute of Agronomy, University of Natural Resources and Life Sciences Vienna, Tulln, Austria; ^5^ Human-Centered AI Lab, Department of Forest- and Soil Sciences, Institute of Forest Engineering, University of Natural Resources and Life Sciences Vienna, Vienna, Austria; ^6^ Verein zur Förderung einer nachhaltigen und regionalen Pflanzenzüchtung, Zwettl, Austria

**Keywords:** drought tolerance, GWAS, MAS, plant breeding, winter wheat, XAI, UAV remote phenotyping, smart agriculture

## Abstract

Marker-assisted selection (MAS) plays a crucial role in crop breeding improving the speed and precision of conventional breeding programmes by quickly and reliably identifying and selecting plants with desired traits. However, the efficacy of MAS depends on several prerequisites, with precise phenotyping being a key aspect of any plant breeding programme. Recent advancements in high-throughput remote phenotyping, facilitated by unmanned aerial vehicles coupled to machine learning, offer a non-destructive and efficient alternative to traditional, time-consuming, and labour-intensive methods. Furthermore, MAS relies on knowledge of marker-trait associations, commonly obtained through genome-wide association studies (GWAS), to understand complex traits such as drought tolerance, including yield components and phenology. However, GWAS has limitations that artificial intelligence (AI) has been shown to partially overcome. Additionally, AI and its explainable variants, which ensure transparency and interpretability, are increasingly being used as recognised problem-solving tools throughout the breeding process. Given these rapid technological advancements, this review provides an overview of state-of-the-art methods and processes underlying each MAS, from phenotyping, genotyping and association analyses to the integration of explainable AI along the entire workflow. In this context, we specifically address the challenges and importance of breeding winter wheat for greater drought tolerance with stable yields, as regional droughts during critical developmental stages pose a threat to winter wheat production. Finally, we explore the transition from scientific progress to practical implementation and discuss ways to bridge the gap between cutting-edge developments and breeders, expediting MAS-based winter wheat breeding for drought tolerance.

## 1 Introduction

**Figure 1:**
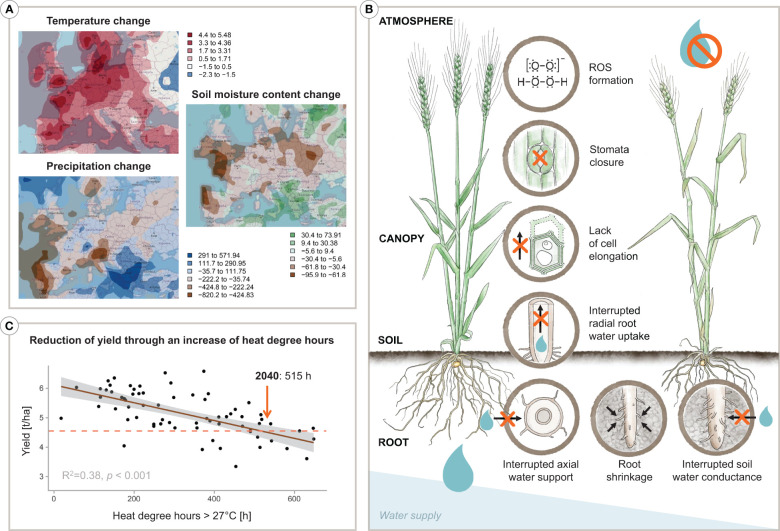
Infographic depicting **(A)** climatic projections by 2060 in Europe based on the SSP1 scenario, **(B)** an increase of heat degree hours results in a decrease of yield in wheat, and **(C)** a plant’s various physiological responses to water deprivation. Specifically, the projections in **(A)** show the change of the annual maximum temperature [°C], the change of precipitation [mm], and the change of the soil moisture content [kg/m^2^] by 2060 relative to 2020 considering the best case SSP1 narrative following the ‘Green Path’. In detail, maps are based on the GFDL-ESM4 model data provided by NOAA-GFDL, release year 2018 (Krasting et al., 2018) representing the SSP1-2.6 model made available through the Coupled Model Intercomparison Project, CMIP (Eyring et al., 2016). Furthermore, in **(B)**, a decrease of wheat yield [tonnes/ha] can be seen with rising heat degree hours over the vegetation period. On-farm yield data and heat degree hours represent averages of six districts in Lower Austria during the years 2002–2014. The dashed line indicates a projection to 2040. An overview of the physiological reactions of a plant to drought stress is presented in **(C)**. Designed by Tatjana Hirschmugl and Eva M. Molin.

Water scarcity is seen as a key threat for the 21^st^ century (Unesco, 2012), with global water demand expected to surpass supply by 40% by 2030 (Gilbert, 2010). Even under the ‘Green Path’ Shared Socioeconomic Pathway (SSP1), which envisions a future with increased sustainability and reduced resource and energy consumption (Riahi et al., 2017), Europe is projected to experience a rise in the maximum annual temperature of over 5°C, a decrease in precipitation of about -700 mm, and a reduction in soil water content of up to -62 kg/m^2^ by 2060 relative to 2020 (see **Figure 1A**). Given that agriculture is the primary user of freshwater, accounting for 70% of total withdrawal globally (FAO, 2010; Hoekstra and Mekonnen, 2012), it is crucial to develop new strategies to enhance crop water use efficiency through agronomy or breeding to tackle the impending water crisis (Sposito, 2013; Turner et al., 2014; Bodner et al., 2015).

The yield of wheat, one of the key staple crops worldwide and particularly in Western Europe (about 14% and 25% of total cropland area respectively; FAO, 2023), has seen a steady increase during the second half of the 20^th^ century. However, this trend has shifted since the 1990s, with yields reaching a peak and partially even slightly decreasing, and showing an increasing variability year-to-year. Brisson et al. (2010) suggested two main factors for this shift: (i) the effects of climate change and (ii) a decrease in input intensity, primarily of N-fertiliser, due to EU agri-environmental regulations. Therefore, future production of key crops like wheat will have to cope with higher resource constraints, in terms of both water and nutrients, even in Europe’s temperate climate conditions. Particularly in sub-humid to semi-arid regions, the balance between soil water supply and crop water demand largely determines achievable yield levels (Lalic et al., 2013). With projected higher temperatures and more unpredictable rainfalls, the frequency of periods of crop water shortage is likely to increase (Qin et al., 2023). Additionally, the co-occurrence of heat and drought is expected to have the most significant impact on wheat yield, with a predicted global reduction of 3.9% (Heino et al., 2023). On a more regional scale, for instance, climate change projections for the Pannonian lowlands, an important wheat-producing region in Europe, indicate that the number of dry days with water deficit during the vegetation period will increase (Trnka et al., 2011; Lalic et al., 2013; Schils et al., 2018; van der Velde et al., 2018). Evaluation of past yield data and simulation model predictions point to a high risk for wheat production under climatic conditions with hot temperatures (*>*25°C; Lüttger and Feike, 2018; **Figure 1B**) and drought occurring at a sensitive developmental stage, such as germination, tillering, flowering or grain filling (Yu et al., 2018; Senapati et al., 2021; Xu et al., 2022). These factors underscore the urgency to speed up the breeding process for more drought (and heat) tolerant varieties to keep pace with the rate and scale of climate change.

One of the methods that has revolutionised plant breeding by improving its efficiency, speed and precision is marker-assisted selection (MAS) (Collard and Mackill, 2008). There are different MAS strategies such as MAS backcrossing, MAS pyramiding or early generation MAS (Jeon et al., 2023), all of which use DNA-based markers to help select lines with the desired traits. The limited number of markers per trait and its restricted use for traits under complex genetic control are major limitations of MAS. These limitations led to the development of other marker-based strategies such as genomic selection (GS) or crop growth models (Budhlakoti et al., 2022; Zhang et al., 2022). Unlike MAS, GS uses all available (genome-wide) markers to calculate a breeding value and has been shown to outperform MAS in several studies (Arruda et al., 2016; Degen and Müller, 2023). Despite these advancements, MAS is still extensively used to efficiently screen for traits of interest. For instance, MAS has been employed in wheat breeding to improve resistance to biotic and abiotic stresses and to maintain yield potential (Song et al., 2023; Subedi et al., 2023). A notable advantage of MAS may be that, compared to the genome-wide approach of GS, only a few markers ultimately need to be used by the breeder, making MAS –despite its limitations– an affordable solution for practical breeding.

However, for a MAS programme to be successful, certain prerequisites must be met: the generation of high-quality phenotypic and genotypic data, the understanding of marker-trait associations, the characterisation of reliable markers and, finally, the development of cost-efficient and easy-to-use genotyping approaches. In this review, we therefore attempt to cover this process using the example of winter wheat breeding for increased drought tolerance. As a starting point, (i) we revisit the physiological mechanisms and corresponding traits that have been associated with drought tolerance in winter wheat under different drought regimes (Section 2), (ii) we further discuss traditional and modern phenotyping approaches focusing on airborne technologies and time series records and provide a guide for airborne data acquisition for winter wheat (Section 3), (iii) we include genome-wide association studies (GWAS), an important computational approach that links the recorded phenotypes with the genotypes for the identification of genetic markers used in MAS (Sections 4 and 5), and finally, (iv) we address artificial intelligence (AI) models accompanied by explainable AI (xAI) methods that could support the breeding process at several steps in the context of smart agriculture (Section 6). Attempting to bridge the gap between scientific innovations and their application in practice, (v) we conclude this review with an overview of the practical work of plant breeders (Section 7) and where these (novel) cutting-edge approaches could fit in and help accelerate the breeding process.

## 2 Physiological mechanisms underlying drought tolerance

Historically, advances in wheat breeding have largely been driven by increased yield potential through better assimilate partitioning towards grain sinks, sustained by prolonged assimilate source activity due to extended green canopy duration (Lichthardt et al., 2020). However, under water-limited conditions, yield formation is a complex function of total water uptake, water use efficiency, and harvest index (Passioura, 1977). Ecophysiological theory has guided trait-based breeding by uncovering stress adaptation strategies in natural vegetation. Levitt’s scheme of dehydration avoidance, dehydration tolerance, and drought escape (Levitt, 1980) serves as a guiding framework for physiological breeding: plant traits underlying individual stress response types aid targeted selection for crop adaptation in water-limited environments (e.g., Richards, 2006; Araus et al., 2008; Cattivelli et al., 2008).

The selection of relevant traits involved in drought tolerance mechanisms that could potentially lead to better and more stable yields strongly depends on the time when the drought occurs (van Ginkel et al., 1998; Blum, 2011). For instance, the phenological adaptation (‘drought escape’) of early maturity might be especially sensitive to early drought events while thriving in summer-dry regions with water deficiency during the grain-filling stage. Dehydration avoidance by ‘water saving’ (Levitt, 1980) might result in suboptimal use of available water under moderate drought regimes, while in situations with more severe drought and crop growth largely dependent on stored soil moisture from off-season rainfalls, a ‘conservative’ water use preserves water for grain filling and yield formation (Mori et al., 2011).

As highlighted in **Figure 1C**, the regulation of plant water balance forms the physiological basis for identifying potential breeding traits for more drought-tolerant plants. Whether transpiration can meet the potential demand, driven by the atmospheric vapour pressure deficit, depends not only on the availability of soil water but also on the transport capacity of soil and plants under variable driving gradients (e.g., Maseda and Fernandez, 2006). In coarse to medium-textured soils, the transport of water through the tortuous soil pore system to the root surface drops sharply when larger pores drain upon successive soil drying, resulting in supply limitation (wilting) at a water content substantially higher than the permanent wilting point (Czyż et al., 2012). With successive drying, the root-soil contact can be lost due to root shrinkage and air gap formation as well as root mucilage becoming hydrophobic to protect root tissues from dehydration (Carminati and Vetterlein, 2013; Affortit et al., 2023). Stomata are the ultimate regulators of crop water transport, providing a mechanism to prevent plants from dehydration damage (cf. **Figure 1C**). Stomata thus act upon imbalances between vapour losses from and liquid water transport to the transpiring leaves. Root water uptake (Abdalla et al., 2022) and xylem transport (Cruiziat et al., 2002) are crucial for stomata regulation, mediated by chemical and hydraulic signals within plant-specific safety margins (Sperry and Love, 2015). Sustained xylem water flow under high-pressure gradients between soil and atmosphere without interruption of transport vessels by air embolism, leading to an eventual hydraulic failure of the transport system, has been suggested as one of the key bottlenecks for crop performance in dry environments (Sperry et al., 1998; Vadez et al., 2013; Vadez, 2014). Plants relying on high safety margins with sensitive stomata response to tissue dehydration (isohydric behaviour; Tardieu and Simonneau, 1998; Hochberg et al., 2018), also have to cope with increased leaf temperature and high radiation load at the leaf, which leads to an overproduction of reactive oxygen species that cause metabolic disorders and limit plant growth and development (Mukarram et al., 2021). Within this general framework of physiological mechanisms and related traits, Blum (2009) points to maximising water uptake as a focus for breeding because it is generally compatible with high yields, i.e. genotypes that fall into the category of ‘water wasters’ according to Levitt’s framework. Efficient water uptake by the root system is a desirable breeding objective (Vadez et al., 2007). In wheat, physiological and root research studies indicate a significant contribution of the root system to increased drought tolerance (e.g. MansChadi et al., 2008; Palta et al., 2011; Becker et al., 2016; Li et al., 2021).

To expand the germplasm sources of (novel) stress tolerance traits, landraces and crop wild relatives are a valuable resource offering a wealth of diversity (Galluzzi et al., 2020) that could be transferred into breeding programmes, as has been extensively reviewed for wheat (Valkoun, 2001; Reynolds et al., 2006; Trethowan and Mujeeb-Kazi, 2008; Nakhforoosh et al., 2015; Lehnert et al., 2022; Aloisi et al., 2023; Shokat et al., 2023). Specifically, cereal genetic resources could contribute to improved drought tolerance through higher water use efficiency (Konvalina et al., 2010), rapid early development (Mullan and Reynolds, 2010), stem reserve demobilisation, osmotic adjustment (Reynolds et al., 2006), and even plant waxiness (Patidar et al., 2023). Several studies also suggest a contribution of root traits (e.g., Reynolds et al., 2006; Sanguineti et al., 2007; Trethowan and Mujeeb-Kazi, 2008; Lopes and Reynolds, 2010; Nakhforoosh et al., 2014).

Despite these studies, further progress in physiological and trait-based breeding to accelerate wheat improvement for future environmental conditions critically depend on adequate selection strategies that combine (advanced) targeted trait phenotyping (see Section 3) with modern genetic tools (see Sections 4 and 5).

## 3 From traditional to airborne phenotyping

The practice of measuring phenotypic traits dates back to Neolithic agriculture when domesticated cereals were intentionally selected for traits such as broad kernels (Zohary et al., 2012). Today, one of the cornerstones of plant breeding is the selection of superior individuals based on phenotypic traits (e.g., grain yield), and more recently, the identification of genome regions controlling these traits (cf. Sections 4 and 5). With advancements in sensor technology, phenotyping has evolved into a high-throughput process, including remote sensing and machine learning (ML), offering solutions for precision agriculture and digital plant breeding (Walter et al., 2015; Pieruschka and Schurr, 2019; Holzinger et al., 2022a; Jeon et al., 2023). This diversity of phenotyping approaches is mirrored in the wide range of data and data formats obtained during the breeding process by different sensors (Thoday-Kennedy et al., 2022), such as visual scorings, direct measurements of plant phenotypic parameters, meteorological readings, and hyperspectral and multispectral measurements (Heremans et al., 2015; Adão et al., 2017; Becker and Schmidhalter, 2017; Hu et al., 2020; Saranya et al., 2023), which we aim to cover in this review with respect to wheat.

### 3.1 Traditional phenotyping

Modern plant breeding still depends on traditional phenotyping, which includes visual scorings, plant measurements, and destructive sampling followed by laboratory analysis (Furbank and Tester, 2011; Atkinson et al., 2018). Each type has unique characteristics in terms of precision and measurement speed. Non-destructive measurements are easily measured, such as plant height and visual assessments of disease occurrence, phenology, and plant architecture. These visual assessments are commonly used and are also applied for official national variety testing, e.g., in Austria (Kumar et al., 2016; Steiner et al., 2017; Anderegg et al., 2020; AGES, 2023; Lunzer et al., 2023). However, the precision of non-destructive measurements can be limited by various factors such as observer variability and lighting conditions. Conversely, destructive measurements involve the collection and analysis of plant samples to acquire data on above-ground dry matter, grain yield, and quality traits like protein content and baking quality. Despite offering high precision, these measurements are time-consuming, destructive, and often limited by cost considerations.

As for breeding experimental setups, they can be classified based on the degree of control over environmental conditions (Hammer and Hopper, 1997). Growth chambers provide highly controlled conditions, where numerous environmental variables such as temperature, light intensity, and *CO*_2_ concentration can be manipulated (Rezaei et al., 2018). Semi-controlled conditions, observed in, e.g., greenhouses and rain-out shelters, offer some control over environmental factors, with greenhouses affording greater control than rain-out shelters (Yadav, 2017; Rezaei et al., 2018). Finally, experiments under field conditions feature the lowest control over environmental variables. Nevertheless, field experiments are undoubtedly relevant, since most of them are conducted in the field under uncontrolled conditions (Hammer and Hopper, 1997). They allow for scientific testing of experimental factors under conditions similar to agriculture practice. Experimental factors can include varying genotypes, sowing times, fertilisation, plant protection, irrigation and disease occurrence due to natural pressure as well as artificial inoculation (Buerstmayr et al., 2000; Koppensteiner et al., 2022).

Observational units vary across setups, ranging from plots in field experiments and rain-out shelters to pots in greenhouses and growth chambers (Buerstmayr et al., 2000; Yadav, 2017; Rezaei et al., 2018; Koppensteiner et al., 2022). In field trials, units of observation include single seeds (Zhu et al., 2012), single rows (Buerstmayr et al., 2000), micro-plots (Miedaner et al., 2006), and large plots (Koppensteiner et al., 2022), e.g., 1.5 m by 7 m, depending on the amount of available seed material of a genotype in the respective stages of the breeding process. In the context of UAV-based sensor systems discussed in this review, micro-plots and large plots are the most relevant observation units. Measurements on more detailed levels are possible depending on the specifications of sensor systems and operational flight height. Despite the significance of field experiments, conducting field phenotyping is arduous, time-intensive, and susceptible to human and environmental variability. Therefore, there is a pressing need to enhance field phenotyping capabilities to facilitate accurate and high-throughput phenotyping, thus expediting crop breeding processes (Yang et al., 2020).

### 3.2 Remote sensing

Remote phenotyping techniques in digital agriculture are prized for their non-destructive nature and their ability to improve data collection accuracy and efficiency (Sishodia et al., 2020; Jeon et al., 2023). These techniques rely on remote sensing, which involves detecting electromagnetic radiation across various wavelengths emitted, reflected, or transmitted by objects. Remote sensing measurements are categorised into direct and indirect methods. Direct measurements involve directly gauging traits of interest, such as plant height using digital surface and terrain models (Holman et al., 2016), while indirect measurements estimate traits using statistical or ML models like biomass and water stress estimates (Wang et al., 2016; Das et al., 2021).

Remote phenotyping can be conducted at various scales: ground-based - handheld or vehicle-mounted (Kumar et al., 2020; Tang et al., 2023), aerial - via aircraft or UAVs (Fei et al., 2023; Nguyen et al., 2023), and satellite platforms like Sentinel-2 (Zhao et al., 2020; European Space Agency, 2023a), Landsat (Zhou et al., 2020; Darra et al., 2023; NASA, 2023), WorldView-2 and 3 (Tattaris et al., 2016; Yuan et al., 2017; European Space Agency, 2023b), or RapidEye (Eitel et al., 2007; European Space Agency, 2024). To contextualise these platforms, key remote sensing features are spatial, temporal, spectral, and radiometric resolutions (Verde et al., 2018). Spatial resolution refers to pixel size, temporal resolution to the time between measurements, spectral resolution to the number of spectral channels, and radiometric resolution to a sensor’s ability to detect varying energy quantities in a specific spectral channel. Each phenotyping platform presents trade-offs; for instance, ground-based techniques offer high spatial resolution but require dedicated manpower, leading to lower time resolution. Aerial technologies offer enhanced operational performance and sub-centimetre spatial resolution (Bhandari et al., 2020) but are weather-dependent, limiting time-series data availability. Satellites provide densely populated time series but sacrifice spatial resolution, with modern satellites offering resolutions as low as 31 cm in the case of Worldview-3 (European Space Agency, 2023b). Moreover, in general, increasing sensor-object distance or increasing the swath width of the satellite, i.e. the horizontal distance covered by a satellite sensor, can improve temporal resolution by allowing the sensor to revisit the same location more frequently. However, this enhancement comes at the cost of diminished spatial resolution (Kadhim et al., 2016). Other trade-offs do not depend on the spatial resolution, but, for UAV, the maximum weight of a payload determines the equipped camera and therefore the spectral resolution available to be measured (Mohsan et al., 2023).

Another key concept in remote sensing and therefore in remote phenotyping is the Ground Sampling Distance (GSD), i.e. the spatial spacing between the centres of two consecutive pixels as measured on the ground. It is determined by several key factors: altitude (*h*), denoting the height above the ground at which the sensor is positioned and affecting the scale of the captured image; sensor size (*s*), representing the physical size of the sensor in the camera, typically measured in mm, larger sensors capturing more detail and impacting the GSD; focal length (*f*), the distance from the optical centre of the lens to the camera sensor, measured in mm, influencing the field of view and magnification of the captured image, and image resolution (*r*). The GSD is mathematically represented as:


$$\text{GSD }\left(\text{m}\right)=\frac{h\;\left(\text{m}\right)\times s\;\left(\text{mm}\right)}{f\;\left(\text{mm}\right)\times r\;\left(\text{pixels}\right)}$$

This metric is important because it directly determines the spatial resolution of the imagery, affecting the level of detail that can be captured and the accuracy of any measurements or analyses conducted on the images. Generally, a smaller GSD indicates a higher spatial resolution and finer detail in the imagery. GSD values vary across different imaging platforms. For UAV imaging, GSD can vary depending on factors like altitude and sensor specifications, generally falling between 0.5 to 10 cm per pixel (Yuan et al., 2018). This range allows for moderately detailed aerial imagery suitable for various agricultural and environmental applications. On the other hand, satellite imaging offers broader coverage but typically lower spatial resolution. GSD for satellite imagery can range from 30 cm to several m per pixel, depending on the satellite platform, sensor, and imaging mode employed (Chawade et al., 2019).

In plant breeding, the field experimental plot is the typical unit of observation (Hammer and Hopper, 1997). While current satellite systems’ spatial resolution may be inadequate for precise phenotypic parameters at a plot level (Tattaris et al., 2016), ground-based and aerial remote sensing approaches offer suitable spatial resolution. UAVs, with their flexibility, extended operational times, lower cost, and high spatial resolution in the low centimetre range, emerge as promising phenotyping platforms for plant breeding and precision agriculture (Sishodia et al., 2020; Guo et al., 2021).

### 3.3 UAV-based remote phenotyping

UAV remote sensing coupled with ML provides a non-destructive method that enables repeated plant measurements over time. This is a significant improvement over traditional methods, which are laborious, time-consuming and expensive (Galieni et al., 2021; Nguyen et al., 2023). Therefore, the use of UAVs for remote phenotyping has become a well-established practise in plant breeding (Yang et al., 2020; Guo et al., 2021). Compared to other remote sensing platforms, UAVs offer several advantages. They are capable of swiftly collecting spectral data, outperforming the speed of handheld devices. They can capture data at a higher resolution compared to aerial cameras operated from a manned aircraft, and they are not dependent on satellite overpasses for data collection in the region of interest (Kim et al., 2019).

#### 3.3.1 An overview of UAV sensor systems

UAVs can be equipped with passive sensors, such as multispectral, hyperspectral and thermal cameras, or active sensors, such as Light Detection And Ranging (LiDAR) (Thoday-Kennedy et al., 2022).

Since multispectral and hyperspectral cameras can capture data at various wavelengths (also outside the visible spectrum), their use in agricultural applications offers many benefits. They can identify and monitor crop health and stress (Yang et al., 2009; Virnodkar et al., 2020), determine and map corn emergence uniformity (Vong et al., 2022) and quickly detect diseases and pests (Prabhakar et al., 2012). Multispectral leaf reflectance data are very useful because they contribute to computing indices widely used in agriculture (see **Table 1** for an overview of the main vegetation indices). Additionally, both multispectral and hyperspectral data can be utilised to estimate crop yields using ML methods (Fei et al., 2023; Joshi et al., 2023). In contrast to multispectral sensors, which typically capture broader spectral bands with spectral resolutions from 10 to 100 nm, hyperspectral sensors offer a much higher spectral resolution, often within 1 to 10 nm (Adão et al., 2017). They effectively capture a spectral continuum across hundreds of contiguous, narrow bands, enabling detailed pixel-by-pixel analysis. Hyperspectral cameras are capable of capturing not only the visible (400-700 nm) and near-infrared (NIR, 700-2500 nm) wavelength ranges but also radiation from the ultraviolet (UV, 100-400 nm) to thermal infrared (TIR, 3000-15000 nm) wavelengths. However, the large data storage required for hyperspectral data can limit its use in large-scale applications (Sun et al., 2019). Therefore, despite their significant advantages, hyperspectral applications in large-scale wheat phenotyping could face challenges related to data storage, management, and budget constraints (Ang and Seng, 2021).

**Table 1:** Most used indices of remote phenotyping applied in wheat breeding.

Name	Formula	Properties	Reference
**NDVI** Normalised Difference Vegetation Index	$\frac{NIR-R}{NIR+R}$	Vegetation density, plant health, and land cover monitoring	(Carlson and Ripley, 1997)
**EVI** Enhanced Vegetation Index	$G_{F}\frac{NIR-R}{NIR+C_{1}R-C_{2}B+L}$	Sensitivity in high vegetation areas and aerosol correction	(Matsushita et al., 2007)
**SAVI** Soil Adjusted Vegetation Index	$\frac{\left(NIR-R\right)}{\left(NIR+R+L\right)}(1+L)$	Vegetation index corrected for Soil Condition	(Huete, 1988)
**NDWI** Normalised Difference Water Index	$\frac{NIR-SWIR}{NIR+SWIR}$	Water presence detection and water content sensitivity	(Gao, 1996)
**LAI** Leaf Area Index	$\frac{-\ln P(\theta )\cos(\theta )}{G(\theta )\Omega (\theta )}$	Green leaf area measurement and ecosystem dynamics monitoring	(Nilson, 1971)
**TCARI** Transformed Chlorophyll Absorption in Reflectance Index	$3\cdot \left(R_{700}-R_{670}\right)-0.2\left(R_{700}-R_{550}\right)\frac{R_{700}}{R_{670}}$	Chlorophyll estimation in vegetation	(Haboudane et al., 2002)
**GNDVI** Green Normalised Difference Vegetation Index	$\frac{NIR-G}{NIR+G}$	Vegetation monitoring	(Rahman and Robson, 2016)
**MSAVI** Modified Soil Adjusted Vegetation Index	$\frac{2NIR+1-\sqrt{{\left(2NIR+1\right)}^{2}-8(NIR-R)}}{2}$	Enhanced sensitivity to low vegetation	(Qi et al., 1994)
**ARI** Anthocyanin Reflectance Index	$ARI=R_{550}^{-1}-R_{700}^{-1}$	Detection of plant pigments	(Gitelson et al., 2001)
**NDRE** Normalised Difference Red Edge	$\frac{NIR-\text{Red}\;\text{edge}}{NIR+\text{Red}\;\text{edge}}$	Measurement of vegetation stress	(Tilling et al., 2007)
**CCCI** Canopy Chlorophyll Content Index	$\frac{NDRE-NDRE_{min}}{NDRE_{max}-NDRE_{min}}$	Measurement of chlorophyll content in the canopies	(Fitzgerald et al., 2010)

The LAI formula presented here is not the only one available. Other methods for computing LAI are referenced in Fang et al. (2019). SAVI/EVI: G_F_ is a gain factor, C_1_ and C_2_ are the coefficients to correct for aerosol influences in the red band and L is the Canopy background adjustment factor. LAI: P(*θ*) represents the canopy gap fraction at the zenith angle *θ* and G(*θ*) is the projection function corresponding to the fraction of foliage projected on the plane normal to the solar direction and Ω(*θ*) is the canopy clumping index. ARI/TCARI: The term RY typically denotes the measurement of the red colour at a wavelength denoted by Y, with the unit of measurement being nanometers (nm). CCCI: *NDRE_min_* and *NDRE_max_* represent the minimum and maximum values of NDRE that have been recorded, respectively.

Thermal imaging, which operates within the broader long-wave infrared (LWIR) wavelengths (from 8 to 15 µm), serves as a valuable tool for detecting plant stress. Thermal measurements can be used to evaluate the transpiration status, plant vigour, and the spread of diseases in wheat cultivars (Mahlein et al., 2012) or, together with measurement of the air temperature, to compute the Crop Water Stress Index (CWSI). This index can then be incorporated as a feature in an ML model to provide insights on canopy head evapotranspiration or to segment image data into temperature areas (Zhou et al., 2021b). Moreover, combining thermal imaging data with other phenotypic traits improves the holistic understanding of plant responses to environmental conditions. This synergy enables researchers, breeders and farmers to make well-informed decisions for optimal crop management and resource allocation (Khanal et al., 2017; Stutsel et al., 2021).

On the other hand, unlike camera-based systems that passively capture reflected, transmitted, or emitted light, LiDAR is an active technique that emits laser pulses and measures the time for these pulses to reflect off objects, providing precise distance and spatial data. This has been particularly useful in wheat breeding for estimating plant biomass and plant height (Hütt et al., 2023). Taking advantage of global navigation satellite systems (GNSS) and laser altimetry, and using GIS software, accurate crop height measurements can be obtained by subtracting a digital terrain model from a digital surface model representing the crop canopy surface (Jenal et al., 2021). Although LiDAR systems typically operate at a single wavelength, combining geometric measurement with spectral information is possible, such as registering multispectral camera images with LiDAR point clouds (Hakula et al., 2023), or using LiDAR systems with individual lasers at various frequencies, e.g., Optech Titan (GEO3D, 2023).

#### 3.3.2 Spectral indices supporting smart wheat breeding

In the context of wheat breeding, an index is a mathematical formula designed to provide a comprehensive representation of various plant traits, physiological states and characteristics (Reynolds and Langridge, 2016). It combines different desired traits into a single numerical value, allowing breeders to assess and compare the overall performance of different wheat varieties more thoroughly (Myneni et al., 1995). The computation of these indices creates a multidimensional profile, enriching the complexity of the breeding problem and providing valuable input for machine-learning approaches. Consequently, indices are crucial tools that enable breeders to make informed decisions, optimise their breeding strategies, and ultimately develop wheat varieties that thrive in a wide range of agricultural and environmental conditions in modern research (Radočaj et al., 2023).

In precision agriculture, vegetation indices are broadly categorised into two main types: broadband and narrowband (Thenkabail et al., 2002). Broadband indices, such as the Normalised Difference Vegetation Index (NDVI) (Rouse et al., 1974), integrate information from relatively wide spectral bands, such as the NIR band. These indices offer a generalised measure of vegetation vigour and health. This approach is efficient and simple, making these indices suitable for large-scale agricultural monitoring and management tasks where rapid assessment is prioritised. In contrast, narrowband indices, such as the Chlorophyll Absorption Ratio Index (TCARI) (Haboudane et al., 2002), target specific narrow spectral bands within the electromagnetic spectrum. These indices focus on precise absorption features related to chlorophyll content, leaf structure, and other biochemical properties. Narrowband indices provide high spectral resolution making them valuable for tasks requiring in-depth analysis of plant health and stress. The choice of using either family of indices depends on the specific physiological traits under investigation.

**Table 1** presents several indices common in remote sensing for wheat phenotyping. The practical rationale behind our selection of these indices is the ease of computing them with standard commercially available multispectral cameras (NIR - 700-2500 nm, RGB - 400-700 nm, SWIR - 2500-3000 nm, Red Edge-700-730 nm) and their recognised impact in assessing the plant water status, general stress condition and phenological traits. Vegetation Indices play a crucial role in assessing various aspects of vegetation health and physiological traits. The Normalised Difference Vegetation Index (NDVI) is widely utilised due to its computational simplicity, facilitating assessments of vegetation density, plant health, and water stress (Condorelli et al., 2018; Hassan et al., 2019; Huang et al., 2021). However, limitations such as computational approximations and instrument inaccuracies can occasionally hinder its effectiveness in evaluating plant stress (Khan et al., 2018).

To address these limitations, several alternative indices have been developed. The Enhanced Vegetation Index (EVI) enhances the vegetation signal in high biomass areas and corrects for aerosol factors (Khan et al., 2018). Additionally, the Soil-Adjusted Vegetation Index (SAVI) and Modified Soil-Adjusted Vegetation Index (MSAVI) correct for soil irradiation in areas with low canopy cover (Prudnikova et al., 2019). The Normalised Difference Water Index (NDWI) detects water presence and sensitivity to water content (Wu et al., 2009). The Green Normalised Difference Vegetation Index (GNDVI) specifically targets green vegetation, utilising the green band instead of red. Furthermore, the Normalised Difference Red Edge (NDRE) emphasises the red edge region of the spectrum instead of the red band. These last two indices correlate with leaf nitrogen content and are used for controlling nitrogen leaf status (Li et al., 2019).

For other physiological traits, specialised indices have been developed. The Transformed Chlorophyll Absorption Reflectance Index (TCARI) estimates chlorophyll content in vegetation and biomass (Wang et al., 2022). The Leaf Area Index (LAI) measures foliage density within a canopy by comparing leaf surface area to ground area. The Anthocyanin Reflectance Index (ARI) identifies the presence of anthocyanins, aiding in the assessment of plant stress, phenology, and disease infection (Koc et al., 2022). Lastly, the Canopy Chlorophyll Content Index (CCCI) estimates chlorophyll levels in vegetation by combining red and red edge bands (Cummings et al., 2021).

Each of these indices offers unique insights that can inform breeding decisions, including assessments of yield potential and drought resistance, thus necessitating careful selection among the myriad indices developed by the remote sensing community (Xue and Su, 2017).

#### 3.3.3 Machine learning for interpreting high-throughput field phenotypic data

In these scenarios, ML techniques showcase their advantage over conventional approaches in predicting phenotypes (Ansarifar et al., 2021). As high-throughput phenotyping methods produce a large volume of data, the use of ML becomes pivotal in accurately interpreting and effectively leveraging this data, leading to more precise phenotype predictions (Shaikh et al., 2022). For example, Wang et al. (2016) presents how random forest (RF) models outperform simple multilayer perceptrons (MLPs) and support vector machines (SVMs) in predicting wheat biomass. Grinberg et al. (2020) provides a comparative study of different ML models on various phenotyping problems across different crops, including wheat. The advent of deep learning enhances the classification of crop images, offering unprecedented granularity in monitoring crop quality, assessing yield, and pinpointing water stress at a pixel-wise level (Chandel et al., 2021). Convolutional neural networks (CNNs) further boost the model’s capabilities, automatically extracting key features and patterns to make reliable phenotype predictions (Jiang and Li, 2020). Moreover, deep learning models have expanded the range of possible predictions to include disease detection, stress severity quantification, and yield (Mohanty et al., 2016; Giménez-Gallego et al., 2019; Zhou et al., 2021a). An intriguing direction that research has taken is semi-supervised approaches to the learning problem (Tang et al., 2023; Zhou et al., 2023). Semi-supervised deep learning is an ML paradigm where a model is trained using a combination of labelled and unlabelled data. It uses the limited labelled data to guide the learning process and improve the model’s performance on tasks such as classification or regression, while also benefiting from the larger pool of unlabelled data for generalisation and enhanced feature representation (Yang et al., 2021). Deep learning significantly improves the model’s ability to generalise and enables accurate and reliable phenotyping models for high-throughput approaches. However, a key drawback of deep learning approaches is that each solution needs to be tailored to the data and the phenotypic trait under investigation.

While traditional methods continue to hold their merits, integrating (UAV-based) remote sensing coupled with ML in phenotyping processes might be essential to obtain better and more resilient crop varieties (Yang et al., 2020). In addition, operational costs could be significantly reduced by cutting fixed costs such as laboratory equipment and workforce. This would lead to improved scalability in the approach and quicker results that are passed over in the data pipeline.

### 3.4 A guide for UAV-based data acquisition for winter wheat

**Figure 2:**
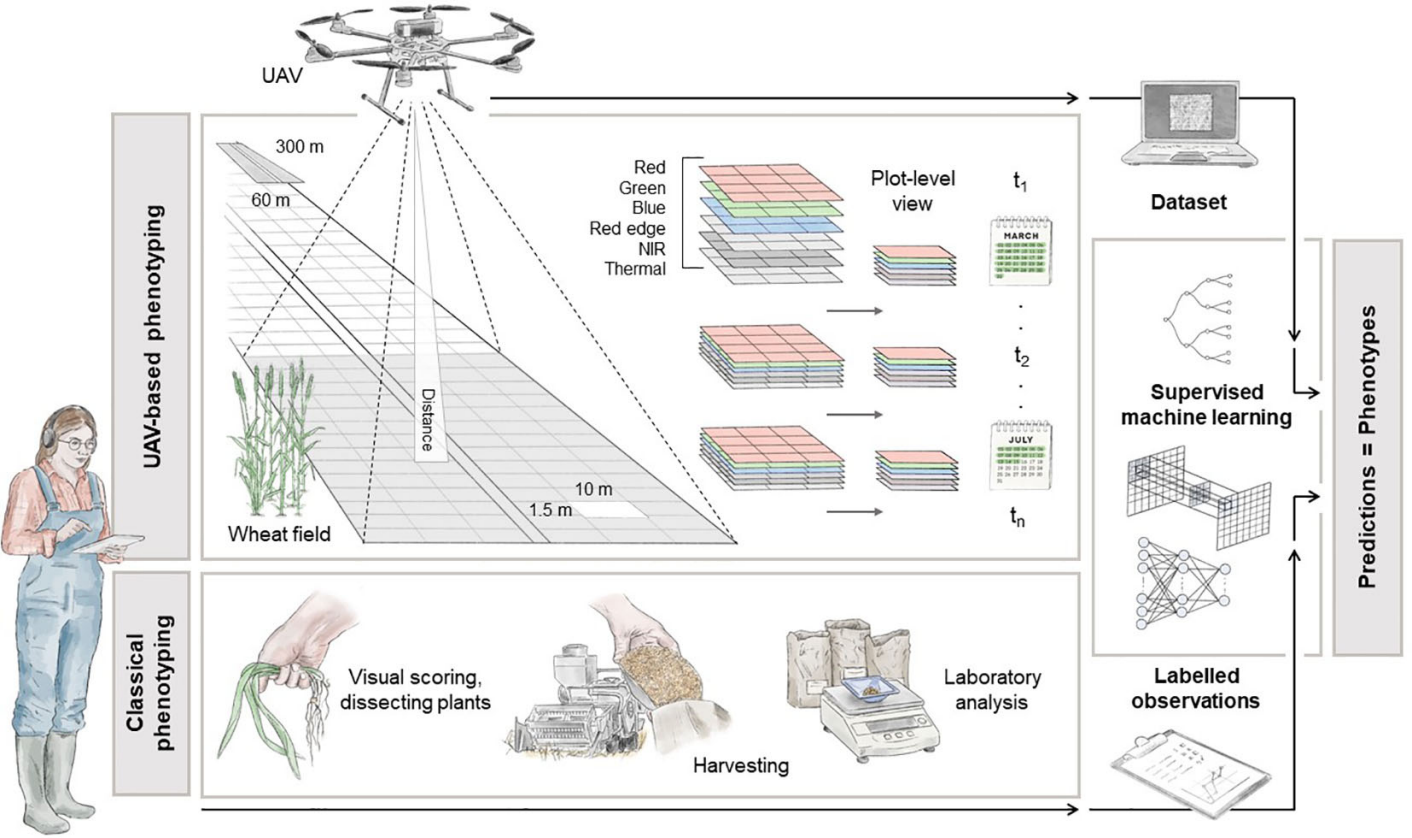
Illustration of a high-throughput UAV-based phenotyping configuration for plot-level analysis. The pipeline explains how to get from the raw data, *in-situ* phenotype acquisitions and raw images, to a structured and cohesive dataset to be employed in ML operations. The depicted dimensions, captured wavelengths, ML models, and traditional phenotyping methods serve as reference points. Designed by Tatjana Hirschmugl.

Moving to the next stage, this Section presents a detailed overview of a potential high-throughput field phenotyping system specifically tailored for winter wheat. The main objective is to facilitate the acquisition of phenotypic data for GWAS (see Section 4) and MAS techniques in the frame of precision agriculture. A schematic representation of the key components of the pipeline is presented in **Figure 2**.

In this scenario, the fundamental premise revolves around the division of the test field into georeferenced experimental plots, overseen by experts tasked with gathering *in-situ* data. This experimental arrangement mirrors established methodologies seen across various research endeavours, aimed at facilitating controlled crop cultivation (Bai et al., 2016; Haghighattalab et al., 2016; Volpato et al., 2021). The grid structure delineating individual plots within the field is visually represented in **Figure 2**. Typically, experts conduct assessments and record measurements by visually inspecting these plots, as demonstrated in Koc et al. (2022). It’s also advantageous to conduct these measurements at specific intervals, tailored to the trait being studied. For instance, in Fernandez-Gallego et al. (2020), multispectral images were captured under direct sunlight on three dates: June 6^th^, 25^th^, and July 3^rd^, 2018, in Belgium, corresponding to the developmental stages flowering and ripening, to monitor wheat ear development and count. During each scheduled flight mission, a UAV systematically follows a predefined grid pattern, meticulously gathering data while traversing the agricultural field.

The UAV could be equipped with a camera capable of capturing a range of spectral information, including RGB, panchromatic, Red Edge, NIR and thermal measures, during its flight (Holman et al., 2016; Tattaris et al., 2016; Duan et al., 2017). The specific selection of spectral bands depends on the particular index to be computed, which in turn depends on the trait under investigation. Additionally, the camera must undergo radiometric calibration to ensure the acquisition of physically meaningful measurements. The spatial resolution of the data acquired is influenced by both the altitude of the UAV and the intrinsic parameters of the camera used. For example, a standard multispectral camera (e.g. AgEagle Aerial Systems Inc, 2023) with 3.2 MP captures images with 2.5 cm GSD at an altitude of 60 m above the ground. The collected data is typically processed using photogrammetric software like Pix4D or Agisoft (Zhu et al., 2019). These software applications are used to create a reflectance map of the agricultural field by orthorectification and stitching individual images to reconstruct a high-resolution representation of the target area. Subsequently, plot-level spectral information is extracted using geospatial software, e.g. GIS, and organised for easy access (Beltrame et al., 2024). This data is then linked with specific plots and expert-acquired labelled information (lower part of **Figure 2**) to create tuples for subsequent ML analysis. These calibrated, cleaned, and standardised datasets can be used in classical preprocessing operations, including image normalisation, data augmentation, and sub-/oversampling techniques. To fully harness the information-rich content obtained, it is essential to select models that can handle the spatial complexity inherent in high-resolution images. For instance, a basic deep learning architecture, such as CNNs, can be used to extract feature maps from images and make accurate phenotype predictions (Kattenborn et al., 2021; Nguyen et al., 2023).

Recent advancements in image analysis, data extraction, and augmentation (Shorten and Khoshgoftaar, 2019), coupled with innovative artificial image synthesis techniques (Lu et al., 2022), and transfer learning (Hutchinson et al., 2017) are greatly enhancing the development and the integration of remote sensing technologies in agriculture. These advancements are starting to contribute to overcoming the phenotyping bottleneck (Song et al., 2021) and significantly enhance the provision of high-quality phenotype data to genotype - phenotype association studies ultimately resulting in an efficient and reliable MAS.

## 4 GWAS - a playground for the identification of genetic markers

Besides a meticulous recording of phenotypic data, MAS depends on the availability of genetic markers linked to the phenotypic trait of interest. Identifying these genetic regions associated with a phenotype is often not a straightforward task: many traits are polygenic, which adds to the complexity of their relationship with the phenotype (Korte and Farlow, 2013; Boyle et al., 2017; Mills and Rahal, 2019; Pierce et al., 2020). The general approach of linking genetic regions to traits, known as genetic mapping, consists of two main strategies: (biparental) linkage mapping (LM) and association mapping (AM) (March, 1999). LM utilises closely related individuals to study the co-segregation of markers and traits due to physical proximity, while AM uses diverse, unrelated populations to detect statistical associations between markers and traits. AM, also known as linkage disequilibrium mapping, exploits linkage disequilibrium (Mackay and Powell, 2007), which is the nonrandom association pattern between alleles at different loci within a population (Nordborg and Tavaré, 2002; Gaut and Long, 2003). Since its introduction to plants (Tenaillon et al., 2001; Thornsberry et al., 2001), AM has become increasingly important in genetic research as cost-effective, high-throughput technologies for genotyping single nucleotide polymorphisms (SNPs) are now available, enabling dense marker coverage (Syvänen, 2005). A particular concept of AM, namely genome-wide association studies (GWAS), has become a common technique for understanding complex traits in plants in general and in many crop species, including wheat (Zhu et al., 2008; Cortes et al., 2021).

The primary advantage of GWAS is that it tests thousands to millions of genetic variants (e.g., SNPs) of many individuals from different populations on a genome-wide scale, allowing more complex genotype-phenotype relationships to be explained than with LM. However, for a genome-wide analysis, the knowledge about and the characterisation of SNPs is an essential part and is driven by the sequencing of the whole genome of the target organism. In the case of wheat, its genome was fully sequenced in 2018 (Appels et al., 2018) and has been continuously improved since then Shi and Ling (2018); Guan et al. (2020); Gao et al. (2023), including the creation of a pangenome (Montenegro et al., 2017; Jayakodi et al., 2021), which provides a valuable knowledge base for the development of a variety of high-density SNP arrays for high-throughput genotyping (Wang et al., 2014; Rimbert et al., 2018; Sun et al., 2020). Finally, to link these genotypic traits to the measured phenotypes, a wide range of GWAS-based tools and statistical methods are available, which have already been used in wheat, as shown in **Table 2**, which are described in the following Section in more detail.

**Table 2:** Common GWAS tools and methods, and examples of their application in wheat.

GWAS tool	Tool reference	GWAS method	Application in wheat
BayesC*π*	Habier et al. (2011)	Bayesian GWAS	Zhao et al. (2013)
BLINK	Huang et al. (2019)	Bayesian GWAS	Devate et al. (2022)
EMMAX	Kang et al. (2010)	Efficient mixed model	Daba et al. (2018) Li et al. (2022)
farmCPU	Liu et al. (2016)	Multiple loci LMM	Gahlaut et al. (2021) Rahimi et al. (2023)
GAPIT	Lipka et al. (2012) and Tang et al. (2016)	Compressed mixed linear model based genomic prediction	Qaseem et al. (2019) Bennani et al. (2022)
MA	Zhou and Stephens (2012)	Genome-wide efficient mixed model	Wu et al. (2021) Ma et al. (2022)
JMP Genomics	SAS Institute Inc (2013)	GLMs	Gizaw et al. (2018)
PLINK	Purcell et al. (2007) and Chang et al. (2015)	Mixed model GWAS	Gogna et al. (2023) Zhao et al. (2023)
SNPtest	Marchini et al. (2007) and Marchini (2010)	Imputation based GWAS	Manickavelu et al. (2017) Muhammad (2021)
sommer	Covarrubias-Pazaran (2016)	Mixed model GWAS	Vukasovic et al. (2022) Dallinger et al. (2023)
TASSEL	Bradbury et al. (2007)	Generalised models and mixed linear models	Lehnert et al. (2018) Akram et al. (2021)

### 4.1 GWAS modelling strategies

The modelling strategies underlying GWAS are diverse from a statistical perspective, of which linear and Bayesian models are the prevailing strategies. Linear models fit linear equations to the data (genetic and phenotypic data), testing each specific marker and its relationship with the phenotype independently, simplifying the computational complexity that could arise from the genetic intricacies in the data (Sabatti, 2013). Generalised linear models (GLMs), as described in Nelder and Wedderburn (1972), add an additional layer of complexity, including a link function to relate input and output, thus providing certain flexibility from the rigidity of linearity. Linear mixed models (LMMs) represent another logical extension of linear models for GWAS and are widely applied (cf. **Table 2**). LMMs include fixed and random effects to model phenotypes, and can account for confounding factors such as population stratification, family structure, etc (Alamin et al., 2022). LMMs also offer versatility as they can analyse many experimental designs (Yang, 2010). These models, as their name suggests, assume a linear relationship between genotype and phenotype. They also assume that the random effects are normally distributed and that there is homoscedasticity in the variance of their errors (Warrington et al., 2014). These are the two main concepts use for GWAS methods based on linearity.

Bayesian models have also been developed and used for GWAS (cf. **Table 2**), fitting all markers simultaneously while addressing the issue of data dimensionality, making them well suited for polygenic traits (Fernando and Garrick, 2013; Miao et al., 2019). These methods require the specification of prior distributions, allowing knowledge of the data to be incorporated into them to yield more accurate results, with the caveat that deviation from the specified distribution can impair performance and statistical power (Cortes et al., 2021). Bayesian GWAS aim to identify sections of the genome that explain more than a threshold of the variance (Fernando and Garrick, 2013; Cortes et al., 2021). The multiple methods developed assume different distributions for the calculation of the priors, having different performance according to the deviation from their actual distribution. Markov Chain Monte Carlo algorithms have been used to infer model parameters using Gibbs-type processes, as in Habier et al. (2011). The posterior probabilities of association, the odds of a specific SNP being actually related to the trait, can be calculated from the Bayes factor (Stephens and Balding, 2009).

### 4.2 Understanding the limitations of GWAS

Despite all these advancements, GWAS still have significant limitations in their design and application (Korte and Farlow, 2013; Wray et al., 2013; Tam et al., 2019; Cortes et al., 2021): they can be limited to the populations that are more represented in the studies, and there can exist a lack of transferability, as results may not extrapolate to other groups (Bouaziz et al., 2011), or the number of ostensible causal variations might be reduced if data from genetically diverse populations were used, so it is paramount to have an adequate representation of the population to reduce the possible biases that can arise from this (Clyde, 2019; Uffelmann et al., 2021). In addition, at this point, the causality or functionality of the linked SNPs is still elusive and only can be validated empirically through further experimentation (Hazelett et al., 2016; Gallagher and Chen-Plotkin, 2018). Non-normality of the data can also be a significant factor that increases error and reduces statistical power (Yoosefzadeh-Najafabadi et al., 2022). When applying GWAS, the risk of finding spurious correlations is ever-present, thus careful consideration must be taken into the model to correct when working with complex traits (Ball, 2013).

As the complexity of genetic architecture increases (Boyle et al., 2017), GWAS methods often fail to identify all genetic polymorphisms that have an effect on the phenotype. This phenomenon, known as missing heritability (Brachi et al., 2011), occurs when the genotype identified with these statistical methods does not fully explain the target characteristics. Missing heritability is thought to be caused partially by polymorphisms that have a small correlation with the target trait, and thus not being significant after Bonferroni correction (López-Cortegano and Caballero, 2019). Bonferroni correction is a method of adjusting *p* values when conducting multiple simultaneous tests on the same dataset; it involves dividing the initial *p* value by the number of hypotheses tested. In the context of GWAS, the relationship between specific SNPs and the desired trait is considered a comparison, so the *p* value is divided by the number of SNPs in the data (Napierala, 2012; Tam et al., 2019). However, Bonferroni correction has its drawbacks, for instance, when dealing with skewed phenotypic data (John et al., 2022). Since many GWAS methods are based on linear regression models, missing heritability could also be addressed with non-linear models (Peng, 2020). Nonetheless, some missing heritability might still be due to an underestimation of the effect sizes of common alleles, unidentified common and rare alleles, epigenetic changes, or in some cases, it might not even be found within genetic information (Marian, 2012; Bourrat et al., 2017). Colinearity is another potential source of reduced efficiency and statistical power for GWAS methodologies, and new strategies are needed to mitigate this limitation (Zhang et al., 2019). Finally, another important limitation of GWAS is high dimensionality of the data (*n*≪*p*), where the number of features (e.g. SNPs) is much larger than the number of cases (e.g. genotypes), a common issue with biological data (Ramstein et al., 2019). Several AI concepts have been applied to overcome these limitations and disadvantages of GWAS (Szymczak et al., 2009; Nicholls et al., 2020; Enoma et al., 2022), some of which already include certain explainability (e.g., Mieth et al. (2021), see also Section 6).

Many target traits of GWAS are highly quantitative and complex. Grain yield and drought stress tolerance, for instance, are affected by interactions between underlying component traits (Allard and Bradshaw, 1964; Hammer et al., 2006). In Section 2, for instance, a wealth of physiological mechanisms that influence drought stress tolerance are presented. These interactions, however, can be non-linear (Chang and Zhu, 2017), which is a relevant challenge in GWAS. In this context, Technow et al. (2015) proposes to incorporate a crop growth model (CGM) directly into genomic analysis. Crop growth models can simulate biological and physical processes in agricultural systems including plants, environment and management (Holzworth et al., 2014). Relevant CGMs in this context need to include genotype-specific parameters (Oliveira et al., 2021). As a result, these models can capture the effects of non-linear interactions between underlying component traits on target traits (Technow et al., 2015). Gu et al. (2014), for instance, applied QTL mapping and the crop growth model GECROS to investigate the effect of genetic variation in leaf photosynthetic rate on crop biomass in rice. Furthermore, CGMs can help in identifying ideotypes to improve target traits and suitability to specific weather and management conditions (Chang et al., 2019; Bogard et al., 2021). Collins et al. (2021), for example, investigated drought adaptation in Australian wheat using the crop growth model APSIM and suggests limited-transpiration rate at high evaporative demand as a promising trait for selection by breeders.

## 5 GWAS to dissect drought tolerance in wheat

Despite its limitations, GWAS has become a crucial method for discovering loci for traits of interest, as discussed in the previous section. Drought is one of the most important abiotic stressors affecting wheat yield (Heino et al., 2023), prompting scientists and breeders to identify loci associated with drought stress tolerance.

In addition to grain yield *sensu stricto*, numerous other drought stress-related traits have been studied in wheat, including plant height and root architecture, as well as phenological traits like days to heading, anthesis or maturity (Mwadzingeni et al., 2016; Molero et al., 2019; Khadka et al., 2020; Saini et al., 2022). A summary of selected characterised markers and their associated traits in the context of drought tolerance in wheat, including the GWAS method used, is given in **Table 3** and will be further detailed in the subsequent sections.

**Table 3:** Selected markers related to drought tolerance in wheat found with GWAS.

Selected trait (s)	Drought during	N° of Markers found	Important markers	Method (tool)	Reference
Leaf chlorophyll content	seedling stage	28	IWB26948	LMM (GAPIT)	Maulana et al. (2020)
Days to wilting	seedling stage	104	WPT-2356	LMM GLM (TASSEL)	Ahmed et al. (2021)
Grain yield and biomass	whole season	73	wsnp_Ex_Rep_c67786_66472676	LMM (GAPIT)	Bennani et al. (2022)
Grain yield	whole season	94	IWA5483	GLM (JMP Genomics)	Gizaw et al. (2018)
Grain yield	whole season	192	IAAV619, wsnp_Ex_c11120_18022932	LMM (TASSEL)	Suliman et al. (2021)
Grain yield	whole season	61	M7661	LMM GLM (TASSEL)	Akram et al. (2021)
Grain yield	whole season	37	M9766, M9769	Compressed LMM (GAPIT)	Mathew et al. (2019)
Grain yield	whole season	45	S7A_112977027	FarmCPU (NA)	Bhatta et al. (2018)
Grain yield	whole season	136	wsnp_BM134363A_Ta_2_4	LMM (GAPIT)	Qaseem et al. (2018)
Stress tolerance index	whole season	9	AX-111169510	LMM PCA + K GWAS (GAPIT)	Zhao et al. (2023)
Days to maturity	whole season	37	M1433, M6472, M1576	Compressed LMM (GAPIT)	Mathew et al. (2019)

### 5.1 Introduction to developmental stages and yield components in wheat

**Figure 3:**
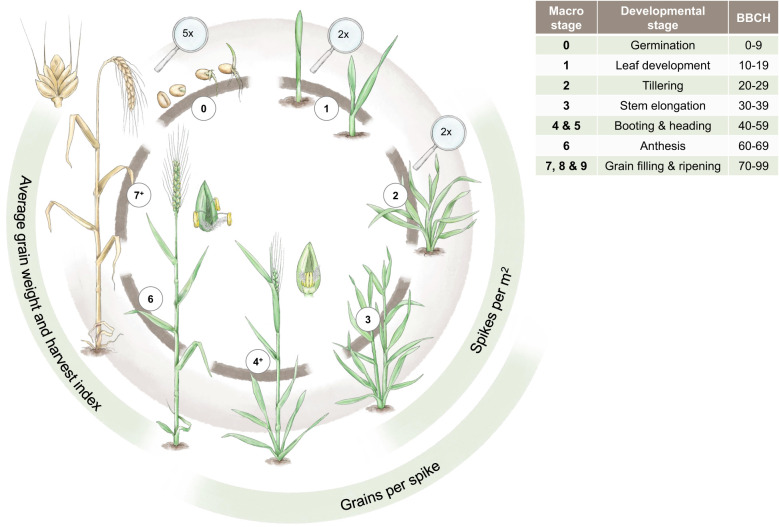
The main developmental stages of winter wheat from germination to ripening are depicted. These developmental stages are contrasted by the most important yield components (spikes/m^2^, grains/spike, and average grain weight and harvest index) as well as the BBCH classification in the adjacent table. The magnifying glasses indicate a magnification for the respective drawings. Designed by Tatjana Hirschmugl.

To characterise marker-trait associations (MTAs) in the context of drought, it is essential to understand the developmental stages of wheat and to know at which stage drought can impact the traits of interest that might also affect grain yield, e.g. yield components, as highlighted in **Figure 3**. For the classification of the developmental stages, we use the commonly applied BBCH-code (Hack et al., 1992). Yield components are generally targets of high importance in plant breeding (Araus et al., 2008). In cereals, grain yield is described as the number of grains per m^2^ multiplied by the average grain size. The number of grains per m^2^ can be further differentiated into the number of spikes per m^2^ and the number of grains per spike. Spikes per m^2^ and grains per spike are established during the vegetative stage before anthesis, while the average grain size is mainly determined later during the generative stage (Geisler, 1983).

The number of spikes per m^2^ is the first yield component determined during plant development. During the tiller differentiation process (BBCH 20, tillering stage, cf. **Figure 3**), the maximum number of tillers is established. Transitioning from BBCH 20 to BBCH 30 (stem elongation stage, cf. **Figure 3**), the number of established tillers is reduced to productive, spike-bearing tillers. Both the differentiation and reduction process of tillers are affected by drought stress. The tiller reduction process, however, is much more sensitive to water shortage than the respective differentiation process (Geisler, 1983). The differentiation process of generative organs, e.g., grains, can be divided into the establishment of spikelets and florets, whereby the primordia of spikelets are already developed by the end of tillering stage (BBCH 20). During stem elongation (cf. **Figure 3**), most spikelets and florets differentiate, and the maximum number of spikelets and florets is present at the beginning of BBCH 50 (heading). Afterwards, reduction processes of spikelets and florets occur until anthesis. The developmental stages from heading until anthesis are especially sensitive to drought stress González-Navarro et al. (2015). If drought stress is too severe, shedding of fertilised florets can occur after anthesis. Furthermore, insufficient water supply can also shorten the period for spikelet differentiation and thus reduce the number of spikelets per spike. In comparison to the simultaneous tiller reduction processes during stem elongation stage, this effect is minor (Geisler, 1983). Starting with anthesis (BBCH 60), the differentiation process of the caryopsis (the grain) occurs, which determines average grain weight (cf. **Figure 3**). In general, the longer the grain filling period during the stages of grain development and ripening (BBCH 70 and 80), the higher the average grain weight is (Ozturk et al., 2006; Klepeckas et al., 2020). The duration of this phase, however, is highly affected by environmental conditions. High temperature and insufficient water supply lead to shorter grain filling periods and thus a low average grain weight and even shrivelled grains (Spiertz, 1974; Klepeckas et al., 2020), as well as a shorter duration for the translocation of assimilates to the grain and thus lowers the harvest index of wheat (Davidson and Campbell, 2011; Neugschwandtner et al., 2015; Koppensteiner et al., 2022).

### 5.2 Markers associated to yield components under drought stress

As the processes of differentiation and reduction for each yield component occur at different developmental stages, they can be significantly affected by temporal environmental conditions (Satorre and Slafer, 2000). For instance, high temperature and water shortages can result in (i) accelerated plant development and consequently shorter differentiation processes for yield components, (ii) more intense reduction processes of individual yield components, and (iii) decreased photosynthetic activity, resulting in fewer available assimilates for grain filling. Besides environmental effects, yield components generally also depend on the genotype and crop management practices, such as sowing, fertilisation, plant protection, and irrigation (Geisler, 1983).

Numerous MTAs have already been characterised in experiments comparing wheat varieties and their responses to drought (**Table 3**). For example, Mathew et al. (2019) discovered associations between markers and biomass allocation to grain yield. Mwadzingeni et al. (2017) identified 334 MTAs with high confidence for traits under both drought and non-drought conditions. However, these markers explain only 20% of the phenotypic variation, which could be a consequence of the statistical stringency inherent in the methodology. The study found that chromosome 5 in genome D included QTLs related to grain yield, as seen in Quarrie et al. (2005). Among the 29 MTAs found for grain yield, some were located in genes annotated as F-box family protein or Sentrin-specific protease, described to have a potential role in drought stress tolerance (Bhatta et al., 2018). Markers such as *Xwmc273.3* and *Xpsp3094.1* have been used in the context of MAS of the yield-related QTL *Qyld.csdh.7AL* to develop high-yielding drought tolerant genotypes (Gautam et al., 2021). Bilgrami et al. (2020) identified SNPs (IWB39005 and IWB44377) related to the number of fertile tillers and total tillers. Suliman et al. (2021) explored grain yield and found 192 related markers, where 25 highly significant SNPs on chromosome 5A have a notable effect on grain yield, making this chromosome a relevant target for yield improvement under drought conditions. Seedling length, days to wilting, and leaf wilting were analysed in Ahmed et al. (2021), who reported 104 associated markers. Multiple phenotypic traits related to yield were used by Qaseem et al. (2018) for GWAS, resulting in 136 MTAs relevant for winter wheat’s positive response to drought conditions.

### 5.3 Traits associated with phenology under drought conditions

It is well described that each developmental stage has its own specific water supply requirements. If drought occurs during water-sensitive developmental stages (cf. **Figure 3**), such as germination, tillering, flowering, or grain filling (Yu et al., 2018; Senapati et al., 2021; Xu et al., 2022), growth and subsequently yield can be significantly impacted (Khadka et al., 2020). Therefore, the effects of the concurrence of critical phenological stages and drought conditions are critical (Langridge and Reynolds, 2021). Thus, in traditional plant breeding, phenological parameters are measured by expert-assessed visual scorings. Selection based on phenological characteristics is then conducted by investigating the coincidence of critical developmental stages with drought, heat, or other harsh environmental conditions (Sallam et al., 2019). Maulana et al. (2020) describes drought-related MTAs at the seedling stage of wheat (**Table 3**). In addition, drought stress during stem elongation can lead to yield reduction up to 71.52% (Ding et al., 2018). Early vigour, the rapid development of leaf area, has been genetically determined by 41 markers associated either with the NDVI or the projected leaf area, which could be used to select for varieties equipped with early vigour in the future (Vukasovic et al., 2022). Farhad et al. (2023) discovered several QTLs (i.e. QDtb.bisa.2D.4) that significantly relate to a shift in the time until booting (days to booting) towards earlier planting. MTAs on chromosomes 2B, 3A and 3D have been found to be related to the number of days to anthesis (Molero et al., 2019). Utilising genetics to select suitable varieties based on phenology is an important technique to face intense drought events. Understanding the link between genotype and phenology is essential to maximise grain yield in these scenarios.

Although these findings are significant and represent a substantial step towards crop optimisation against drought, there remains a large portion of heritability that is unaccounted for (see Section 4). As a result, multiple markers that could be useful for MAS might have gone unidentified. This missing heritability could be due to multiple testing correction or because the statistical tests assume a different distribution than that present in the actual data (Brachi et al., 2011), needing the development of new methods to tackle these issues.

## 6 Accelerating plant breeding processes with explainable AI

Artificial intelligence (AI) is now applied in many areas of the life sciences, thanks to the significant success of ML and particularly neural networks (NNs) as problem solvers (Holzinger et al., 2023a), which also has been enabled by the constant increase in computing power and resources. AI has already made its way into modern crop breeding, being used in the analysis of the increasing amount of plant image data, as well as in the modelling of GS and GWAS, overcoming some of the limitations of commonly used statistical methods (Harfouche et al., 2019; Jeon et al., 2023; Najafabadi et al., 2023). However, many AI algorithms have their caveats, as they often lack explainability and transparency due to their complex architecture. This is commonly referred to as the ‘black box problem’ (Castelvecchi, 2016), which can ultimately lead to the inability to provide users with explanations for their decisions. The emerging field of explainable AI (xAI) introduces new methods aiming to make AI systems more transparent and understandable (Arrieta et al., 2020; Miller et al., 2022; Holzinger et al., 2022b), laying the foundation for the digital transformation of smart agriculture, and especially plant breeding (Harfouche et al., 2019; Holzinger et al., 2022a).

### 6.1 Introduction into xAI methods

Although numerous xAI methods have been developed, and new ones continue to emerge for various NN architectures, no single xAI method or combination fully explains the decision-making process of the models. Each of them sheds light on a different aspect of the AI model’s computation and many times it has been shown that there is no mutual consent between them, leading to the so-called ‘disagreement’ problem (Krishna et al., 2022). Currently, quality metrics for xAI methods (Doumard et al., 2023; Schwalbe and Finzel, 2023) and benchmarks for its evaluation are being defined (Agarwal et al., 2023) to motivate xAI research in directions that support trustworthy, reliable, actionable and causal explanations even if they don’t always align with human pre-conceived notions and expectations (Holzinger et al., 2019; Magister et al., 2021; Finzel et al., 2022; Saranti et al., 2022; Cabitza et al., 2023; Holzinger et al., 2023c).

xAI methods have a coarse division between post-hoc and ante-hoc methods: the post-hoc ones are applied after the training has produced ‘sufficiently’ good results in terms of performance. For example, local interpretable model-agnostic explanation (LIME) (Ribeiro et al., 2016), which constructs local linear explanation models from the synthetic neighbourhood around the inputs, and Shapley additive explanations (SHAPs) (Shapley, 1952; Staniak and Biecek, 2019; Frye et al., 2020; Gevaert et al., 2023), which use game-theoretic notions to measure how influential features are to the prediction of a model, are procedures that could give scientists an interpretation of the ‘black box’ (Bach et al., 2015; Montavon et al., 2019; Amparore et al., 2021; den Broeck et al., 2022; Holzinger et al., 2022b). Counterfactual explanations, inspired by the work of Judea Pearl (Pearl and Mackenzie, 2018), are defined as all possibilities that deviate from the main course of events. In similar terms, the question ‘what if’ is applicable to counterfactual explanations that aim to provide information about features that, if they had different values, would result in a different output for the classification/regression problem (Sokol and Flach, 2019; Dandl et al., 2020). On the other hand, ante-hoc methods do not consist of individual software components applied after the model has converged and its internal parameters have solidified. Instead, they are models with built-in explainability. Decision trees (DTs) are one of the most representative models in this category and are widely used. They divide the space of possibilities into parts separated by feature ranges, making this method one of the easiest to understand (Safavian and Landgrebe, 1991). Generalised Additive Models go beyond linear and logistic regression, allowing the output to be expressed as an additive combination of pre-specified non-linear functions (Wood, 2004). Typically, the family of B-splines provides a balance between good performance and interpretability since these functions can be considered as individual and non-interacting. Bayesian Rule Lists contain IF-THEN statements in a list that describes the decision of the model (Letham et al., 2015). The Bayesian rule comes with the definition of a Dirichlet prior that specifies the number of pseudo-counts for a probability distribution, which is defined by a human domain expert (Koller and Friedman, 2009; Holzinger et al., 2023b). The posterior distribution is computed by a Bayesian update rule and incorporation of the number of times one observed each output label.

Layer-wise relevance propagation (LRP) (Bach et al., 2015; Montavon et al., 2019) is a propagation-based method that uses the model’s internal decision parameters to redistribute explanatory factors over the layers of the model, reaching the input variables and obtaining how important those are to the prediction and the model. While the computation of relevance of each feature or input component is something that is achieved by other methods, like sensitivity analysis (SA) (Simonyan et al., 2013) or SHAP, LRP uniquely computes both positive and negative relevance values. This is particularly important since the components that have positive relevance ‘speak for’ the result (e.g., the predicted class in a classification task), whereas those with negative relevance denote elements that contain evidence against the prediction and weaken the prediction confidence of the model. While this method is applied after the training of the model is accomplished, it is not entirely agnostic about the internal structure of the model. LRP has different variations for different NN architectures; for example, Long short-term memory (Hochreiter and Schmidhuber, 1997) networks have an adequately adapted LRP variation (Arras et al., 2017) that enables perturbation analysis of the input sequence and correspondingly graph neural networks (GNNs) have GNN-LRP (Schnake et al., 2020; Xiong et al., 2022) that uncovers positively and negatively important graph paths. LRP has been used for uncovering spurious correlations (so-called Clever-Hans phenomena) between the input and the output of an NN and also for clustered explanations with Whole Dataset Analysis (Lapuschkin et al., 2019).

### 6.2 Explainable AI methods for modern plant breeding

The plant breeding process, in its entirety, necessitates a high degree of transparency and explainability. Breeders, for instance, need more than just a predictive value to support their selection of genotypes; they rely on a wealth of information to understand the underlying biology and environmental interactions (Harfouche et al., 2019). xAI can be used to confer these qualities into effective ML models at several steps of the breeding process and in a multitude of ways:

- **Processing of UAV-sourced data:** AI is required to uncover the complex relationships between remotely acquired visual feedback and phenotypical traits (see Section 3.3). This is often a statistically ill-posed problem due to the challenges of replicating exact conditions from one year to another, the high number of external factors, and the cost of acquiring large-scale datasets of carefully measured phenotypical traits (Cheng et al., 2023). This statistical ambiguity can lead to both under- and over- fitting depending on the case. In this context, both ante-hoc and post-hoc xAI methods are important, as exemplified in Srivastava et al. (2022) for winter wheat yield prediction. Ante-hoc methods intervene in the form of strong regularisation, or inductive biases, which limit the space of possible models to those that closely follow a human-defined formulation of the problem. For example, Ge et al. (2023) predict rice distribution using a physically interpretable model trained directly using feature interpretation methods. These heavily regularised models often take the form of simple, interpretable algorithmic bricks that are trained to solve specific sub-problems, such as Tang et al. (2022) who integrate the domain knowledge that edge-detection is important directly into their winter wheat lodging detection architecture. Post-hoc methods serve as a necessary human-in-the-loop validation to counteract the difficulty to acquire enough data for a statistically significant validation. They serve as sanity checks that verify if the features deemed important by the model can be traced back to a physically understandable relationship. Such examples abound both inside (Sun et al., 2023) and outside (Temenos et al., 2022) winter wheat literature.
- **Understanding genotype-phenotype relationships:** AI can assist in unravelling the complex relationship between a plant’s genotype and its phenotype in response to environmental conditions. Especially xAI can identify genetic variants that contribute to these traits, particularly those that have non-linear interactions - something that GWAS cannot do (Santorsola and Lescai, 2023). Feed-forward NNs go beyond association testing and can use several individuals with many SNPs to predict traits with an acceptable performance (Sharma et al., 2020). After the end of the training process, the xAI method DeepLift (Shrikumar et al., 2017) can be applied and computed for each input SNP attribution score that can take both positive and negative values (indicating the direction of contribution to the target variable). The SNPs with the highest attribution values can be thought of as potential causal causes and be investigated further for plausibility although the results of this research show that in cases of highly correlated features, DeepLift can perceive for one and the same model different input features (SNPs) as important. Building on their previous work (Mieth et al., 2016), Mieth et al. (2021) demonstrated that xAI can enhance traditional GWAS methods: NNs combined with statistical testing driven by xAI can provide a robust framework to uncover SNPs that play a decisive role in the classification result of the NN. LRP (Bach et al., 2015; Montavon et al., 2019) computes the relevance of each SNP used in the classification as if they were *p* values used to compute statistically significant associations. This approach surpasses the deficiencies of previous architectures that required Bonferroni correction for false rejections and returns additional as well as weak associations that might be significant. It also reduces the return of an incorrect association (statistical noise). However, the biological plausibility of the newly discovered SNPs needs to be validated, particularly if there are no existing GWAS results for them yet. Epistasis, the non-linear, non-additive interaction between SNPs, is another important component of this relationship. It is often overlooked by classical GWAS methodologies, prompting the development of many techniques to try and dissect it (Niel et al., 2015). Romero (2022) describes an innovative process of extracting this behaviour from iterative RFs trained on this data (Basu et al., 2018). One of the advantages of using RF models is that their own architecture is easily interpretable Pfeifer et al. (2022).
- **Understanding complex interactions:** AI can be utilised for modelling and predicting how certain genotypes would react to conditions like drought. Unlike classical statistical multi-omics methods (Yazdani et al., 2022), AI is an effective tool for deciphering the complex interactions within a plant and those interactions a plant has with its environment, such as soil microbiome, weather, and other plants, which can influence its stress tolerance. xAI can provide insights into the reasoning behind these predictions, enhancing our understanding and facilitating targeted breeding strategies. In Niazian and Niedbała (2020), several cases of genotype-environment interactions (G × E) used by several AI models (having as input the genome sequence and output the phenotype) with their corresponding xAI methods were analysed, uncovering the decisive factors for these interactions (Streich et al., 2020). It is also shown that NNs outperform other AI models performance-wise on these tasks most of the time and the sensitivity analysis applied to the NNs detects the most important input variables for a prediction in different tasks such as assessment and classification of genetic diversity, yield component analysis and indirect selection (prediction), yield stability and G × E interaction, biotic and abiotic stress assessment, classical mating designs, and hybrid breeding programmes (Stein et al., 2022).

Scientific progress is based on understanding and explaining observable phenomena, and this is the advantage provided by the use of xAI. AI has been able to find complex relationships between genotype and phenotype, which could not have been found with other methods. However, it is important to apply these techniques with a higher degree of scientific rigour. Methods such as LRP, LIME, or SHAP are able to provide a deeper understanding of the behaviour of the model, and thus of the biological problem, a prerequisite in modern plant breeding (Harfouche et al., 2019).

## 7 Towards the implementation of modern tools for practical plant breeding

**Figure 4:**
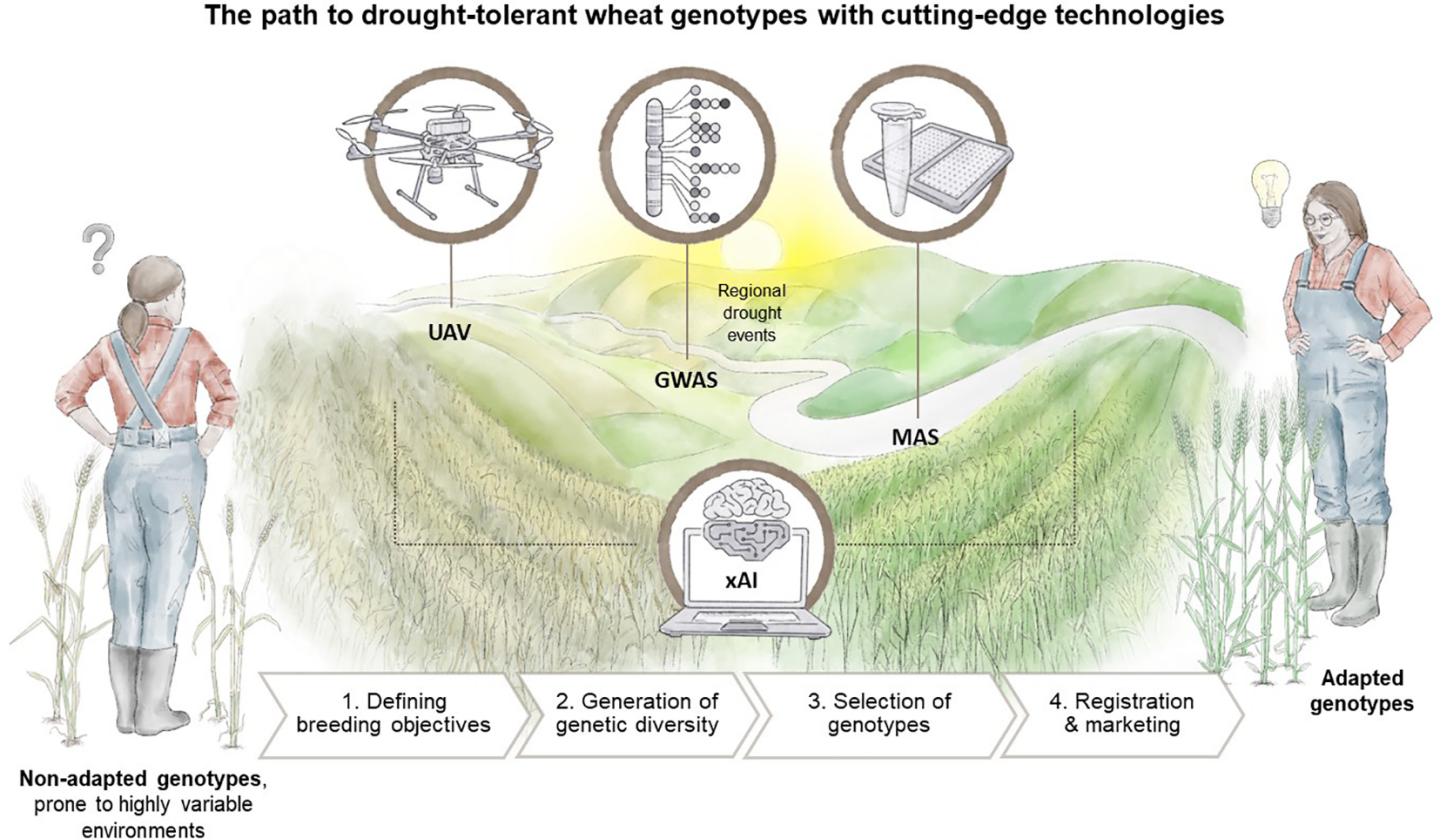
The road to drought-tolerant wheat genotypes remains tedious and time-consuming, but cutting-edge technologies promise to speed up the breeding process. UAV-based phenotyping, GWAS, and ultimately MAS are being increasingly used. However, a cross-process and crucial role is played by xAI, which is not only applied in data analysis and interpretation, but also in decision-making within the entire breeding process, from the definition of breeding goals to the final step of registration and marketing. Designed by Tatjana Hirschmugl.

The previous sections have outlined the advantages of employing modern tools, such as GWAS for genetic marker characterisation, UAV-based remote sensing phenotyping, and the integration of xAI into the breeding process. In this concluding section, we aim to provide an overview of the practical tasks undertaken by plant breeders and how the aforementioned tools can improve the current state of the breeding process, as illustrated in **Figure 4**.

The responsibilities of a plant breeder can generally be categorised into the following steps: (i) defining the breeding objectives, (ii) creating genetic diversity, and (iii) selecting genotypes. Ultimately, a new variety is registered, certified seed is multiplied, and marketed (cf. **Figure 4**). The first step involves identifying key traits that will define a future variety. The second step aims to generate a high genetic diversity, particularly in target traits defined in Step 1, often with limited resources, such as a limited number of crosses or mutagenesis treatments. The third step is centred on the selection of candidate genotypes. This step is heavily reliant on data, necessitating efficient data collection and decision-making, often with limited (financial and human) resources, such as scorings, measurements, samplings and laboratory analysis, as well as downstream data analysis. Consequently, the methods and protocols developed by scientists often need to be scaled down or simplified for easy application within the breeding process.

For example, in GWAS, the ultimate objective is to develop markers taking advantage of a plethora of tools (cf. **Table 2**) with enough precision to predict the presence of a trait of interest. Eventually, these markers (cf. **Table 3**) should be utilised by the breeder, for instance, for screening potential crossing partners (Step 2) and MAS (Step 3). Saini et al. (2022) reviewed, that 86,122 wheat varieties have been analysed with GWAS, resulting in 46,940 loci for various agronomic, physiological, and quality traits. However, their implementation often remains a challenge in many breeding programmes due to several constraints, such as lack of transferability or additional disproportionate costs. Transferability concerns in GWAS are prevalent mostly between different populations and environments, as was shortly discussed in Section 4.2 (Guo et al., 2014; Blake et al., 2020; Mohammadi et al., 2020). Limited transferability due to relevant genotype by environment interactions can be addressed by, e.g., the inclusion of crop growth models (Technow et al., 2015). Mid-range genotyping platforms like KASP™ (Semagn et al., 2014) or MassArray^®^ (Irwin, 2008) offer a relatively flexible, user-friendly, and affordable solution for practical breeding by being capable of screening tens to hundreds of markers in several hundreds of individuals. Both platforms have already been used to design ready-to-use assays for MAS in diverse sets of diploid crop species (e.g., Bomers et al., 2022), and have been successfully applied in polyploid wheat (Bérard et al., 2009; Rasheed et al., 2016; Makhoul et al., 2020; da Costa Lima Moraes et al., 2023; Liu et al., 2023) or aim to do so (Molin, 2024).

The objective of remote sensing phenotyping is to provide fast and precise phenotypic measurements. Particularly for plant breeding, UAV-based phenotyping offers an optimal combination of spatial resolution and speed of measurement (**Figure 2**). This data can be used by plant breeders primarily to enhance genotype selection (Step 3), but also to identify phenotypic diversity (Step 2). Numerous studies have applied UAV-based phenotyping in the context of plant breeding in the past (White et al., 2012; Chapman et al., 2014; Araus et al., 2018; Thenkabail et al., 2018), using a variety of sensors including multi- and hyperspectral, thermal, RGB, and LiDAR to investigate traits such as yield, biomass, plant height, crop health and stress, diseases, pests, as well as nutrient and water content (Yang et al., 2009; Prabhakar et al., 2012; Virnodkar et al., 2020; Zhou et al., 2021b; Thoday-Kennedy et al., 2022; Hütt et al., 2023; Joshi et al., 2023). However, its application in practical breeding is still limited (Matese et al., 2023) due to the need for expertise in several areas, such as drone piloting, legislation, flight planning, photogrammetric processing as well as data processing, modelling, and analysis (White et al., 2012; Chapman et al., 2014; Reynolds et al., 2020; Guo et al., 2021).

In the current scientific dialogue, AI has emerged as a vital tool for problem-solving and knowledge discovery, particularly in the life sciences (Holzinger et al., 2023a). Its applications are manifold and extend to specialised fields like plant breeding. In this context, AI facilitates the analysis of plant image data and plays a crucial role in GWAS and genomic selection (Zhang et al., 2017; Parmley et al., 2019; Aono et al., 2022). AI’s usefulness extends beyond data analysis and permeates the entire decision-making pipeline as depicted in **Figure 4**, from initial data collection and preprocessing (step 1), to feature selection and modelling (step 2), and finally to evaluation and interpretation of results (step 3). The technology’s versatility and computational prowess allow it to process large datasets, discern patterns that may be overlooked by human experts, and provide actionable insights. Essentially, AI acts as a decision support system that enhances the abilities of domain specialists, such as plant breeders, by furnishing them with more accurate and comprehensive information.

The emergence of xAI further enhances the utility of AI in plant breeding. xAI aims to make the complex decision-making processes of AI algorithms transparent and understandable. This is achieved through various methods, such as feature importance ranking, DTs, and counterfactual explanations, among others Holzinger et al. (2021). The increased transparency provided by xAI not only unravels the black-box nature of complex algorithms but also promotes trust and acceptance among human decision-makers. The importance of xAI goes beyond mere understanding of AI’s operations; it addresses ethical and accountability concerns by ensuring that algorithmic decisions can be audited and justified Müller et al. (2022). This is particularly important in high-stakes applications like plant breeding, where decisions can have enduring impacts on agricultural productivity and sustainability. Therefore, the integration of xAI into decision-making processes enhances the trustworthiness and acceptance of AI systems, paving the way for more responsible and effective applications of AI in the life sciences (Holzinger et al., 2022a), including specialised domains such as plant breeding (Harfouche et al., 2019).

In summary, the cutting-edge tools reviewed in this study, encompassing UAV-based phenotyping, GWAS, MAS, bolstered by ML, and the integration of xAI, collectively represent a transformative shift in plant breeding (**Figure 4**). These innovative methods have the potential to revolutionise the way how breeders gather field data, interpret it, and ultimately make informed decisions throughout the entire breeding process, representing a new era in smart agriculture. By leveraging these technological capabilities, breeders can significantly accelerate the development of new crop varieties with improved traits, such as drought tolerance. This acceleration not only reflects the progress in science and technology but also holds the promise of addressing critical agricultural challenges, such as feeding an expanding global population and mitigating the effects of climate change on crop production.

## Author contributions

IC-B: Writing – original draft, Writing – review & editing, Investigation. LJK: Conceptualization, Writing – original draft, Writing – review & editing, Investigation. LB: Writing – original draft, Conceptualization, Investigation, Writing – review & editing. GB: Writing – original draft, Writing – review & editing. AS: Writing – original draft, Writing – review & editing. JS: Writing – review & editing, Writing – original draft. PF-J: Investigation, Supervision, Writing – review & editing, Conceptualization. CS: Supervision, Writing – review & editing. FB: Writing – review & editing. FT: Writing – original draft, Writing – review & editing. MS-Z: Investigation, Writing – review & editing. EZ: Supervision, Writing – review & editing. AH: Funding acquisition, Supervision, Writing – original draft, Writing – review & editing. EMM: Conceptualization, Funding acquisition, Investigation, Project administration, Supervision, Visualization, Writing – original draft, Writing – review & editing.

## Glossary

**Table:**

AI	Artificial Intelligence
AM	Association Mapping
B	Blue band
CCCI	Canopy Chlorophyll Content Index
CMIP	Coupled Model Intercomparison Project
CNN	Convolutional Neural Network
CWSI	Crop Water Stress Index
DT	Decision Tree
EVI	Enhanced Vegetation Index
G	Green band
GIS	Geographic Information System
GLM	Generalised Linear Model
GNDVI	Green Normalised Difference Vegetation Index
GNN	Graph Neural Network
GS	Genomic Selection
GWAS	Genome-wide Association Studies
L	Canopy background adjustment factor
LAI	Leaf Area Index
LIME	Local Interpretable Model-agnostic Explanation
LM	Linkage Mapping
LMM	Linear Mixed Model
LRP	Layer-wise Relevance Propagation
LWIR	Long-wave Infrared
MAS	Marker-assisted Selection
ML	Machine Learning
MSAVI	Modified Soil Adjusted Vegetation Index
MTA	Marker-Trait Association
NDRE	Normalised Difference Red Edge
NDVI	Normalised Difference Vegetation Index
NDWI	Normalised Difference Water Index
NIR	Near-infrared
NN	Neural Network
R	Red band
SAVI	Soil-adjusted Vegetation Index
SHAP	Shapley Additive Explanation
SNP	Single Nucleotide Polymorphism
SVM	Support Vector Machine
SWIR	Shortwave Infrared
TCARI	Transformed Chlorophyll Absorption in Reflectance Index
UAV	Unmanned Aerial Vehicle
XAI	Explainable Artificial Intelligence.
